# Response of the fine root morphological and chemical traits of *Tamarix chinensis* to water and salt changes in coastal wetlands of the Yellow River Delta

**DOI:** 10.3389/fpls.2022.952830

**Published:** 2022-10-11

**Authors:** Jia Sun, Jiangbao Xia, Pengshuai Shao, Jinzhao Ma, Fanglei Gao, Ying Lang, Xianshuang Xing, Mingming Dong, Chuanrong Li

**Affiliations:** ^1^Shandong Key Laboratory of Eco-Environmental Science for the Yellow River Delta, Binzhou University, Binzhou, China; ^2^College of Forestry, Shandong Agricultural University, Tai’an, China; ^3^College of Agriculture and Forestry Science, Linyi University, Linyi, China; ^4^Shandong Hydrology Center, Jinan, China

**Keywords:** coastal wetland, fine root order, groundwater level, morphology, nonstructural carbohydrate, nutrient, water and salt

## Abstract

To explore the adaptation of the fine root morphology and chemical characteristics of *Tamarix chinensis* to water–salt heterogeneity in the groundwater–soil system of a coastal wetland zone, *T. chinensis* forests at different groundwater levels (high: GW1 0.54 m and GW2 0.83 m; medium: GW3 1.18 m; low: GW4 1.62 m and GW5 2.04 m) in the coastal wetland of the Yellow River Delta were researched, and the fine roots of *T. chinensis* standard trees were excavated. The fine roots were classified by the Pregitzer method, and the morphology, nutrients, and nonstructural carbohydrate characteristics of each order were determined. The results showed that the groundwater level had a significant indigenous effect on the soil water and salt conditions and affected the fine roots of *T. chinensis*. At high groundwater levels, the specific root length and specific surface area of fine roots were small, the root tissue density was high, the fine root growth rate was slow, the nutrient use efficiency was higher than at low groundwater levels, and the absorption of water increased with increasing specific surface area. With decreasing groundwater level, the N content and C/N ratio of fine roots first decreased and then increased, and the soluble sugar, starch, and nonstructural carbohydrate content of fine roots first increased and then decreased. At high and low groundwater levels, the metabolism of fine roots of *T. chinensis* was enhanced, and their adaptability to high salt content and low water content soil environments improved. The first- and second-order fine roots of *T. chinensis* were mainly responsible for water and nutrient absorption, while the higher-order (from the third to fifth orders) fine roots were primarily responsible for the transportation and storage of carbohydrates. The fine root morphology, nutrients, nonstructural carbohydrate characteristics, and other aspects of the water and salt environment heterogeneity cooperated in a synergistic response and trade-off adjustment.

## Introduction

The Yellow River Delta (YRD) is an alluvial plain formed by the sediment carried by the Yellow River and deposited in the Bohai Sea depression. It is located at the junction of the river, sea, and land. The ecosystem is unique and has significant ecological functions (Zhao et al., 2020). Due to sea level rises and seawater intrusion caused by global warming, the groundwater level in this region is generally high, with an average groundwater level between 0.5 and 2.5 m, making the shallow groundwater in the YRD more complex and variable than in other areas (An et al., 2013). In areas of the YRD where the groundwater level is shallow, the upwards flow of water from the groundwater to the soil root zone plays an important role in plant growth (Xie et al., 2011). The distribution and evolution of the salinity of the soil in coastal wetlands are closely related to groundwater dynamics, and rising groundwater levels aggravate the degree of soil salinization (Fan et al., 2012). Research has shown that a groundwater level of 3.5 m was the minimum limit to maintain a safe level of soil salinity in the YRD, but at this groundwater level, the decrease in the soil water content exacerbated environmental pressure on *Phragmites australis* growth (Xie et al., 2011). The characteristics of the spatial distributions of soil water and salt were affected by the fluctuations in the groundwater level, and these fluctuations could further affect the growth, development, and succession of vegetation through their root systems.

Fine roots are crucial for the absorption of water and nutrients at both the individual and community levels (Li et al., 2019), and they generally account for 22% to 76% of the net primary productivity of terrestrial ecosystems (McCormack et al., 2015; Ma and Chen, 2016). Moreover, the functional traits of fine roots are commonly considered good predictors of plant adaptation and ecosystem function in response to environmental changes (Freschet et al., 2021). Studies have shown that fine root biomass (FRB) is mainly regulated by the interactions between groundwater and root trait diversity, followed by groundwater, root trait diversity, and soil moisture (Wang et al., 2021a). Although the mean and seasonality of the groundwater depth had substantial effects on FRB, the groundwater affected the FRB mainly through indirect pathways mediated by root trait diversity (Wang et al., 2021a). Important findings of fine root N, P, and N/P ratios were mainly determined by groundwater (Wang et al., 2021b). Salt stress significantly inhibited the growth of absorbing roots (diameter < 0.5 mm), including the root length, surface area, volume, root length density, and the number of root tips, resulting in the inhibition of the growth and development of the whole root system (Li et al., 2021).

Many scholars have studied the structure and function of fine roots from the perspective of root order and found that the same root order in different plant root systems has similar functions, and the root order is directly related to its function (Wang et al., 2006; Withington et al., 2006). Fine roots are classified according to their root order, i.e., the branch position. First-order roots are the most distal, and second-order roots begin at the junction of two first-order roots, and so on, up to fifth-order roots (Pregitzer et al., 2002; Wang et al., 2006; Rewald et al., 2014; Eissenstat et al., 2015). This classification method not only can minimize the internal heterogeneity of fine roots but also can more accurately describe the physiological and ecological processes of underground roots (Pregitzer et al., 2002; Guo et al., 2008). Therefore, studying the relationship between the morphology and function of fine roots from the perspective of root order is of great significance for understanding the heterogeneity within a root system.

Prairie shrubland is a typical type of wetland landscape present in the YRD. The halophyte *Tamarix chinensis* is the most widely distributed shrub species, and it is also the main tree species preferred for the construction of shelter forests in coastal wetlands. *T. chinensis* is a significant component of the YRD wetland ecosystem, and it plays an important role in the process of vegetation restoration and ecological remediation. A study found that, at a high groundwater level, the root topological structure tended to be dichotomous, and the fractal dimension and fractal abundance values were both large. The topological structure of medium and low groundwater level *T. chinensis* tended to be herringbone like, and the fractal dimension and fractal abundance values were small. The *T. chinensis* root system has strong phenotypic plasticity to the heterogeneous water–salt habitat of the groundwater–soil system (Sun et al., 2022). The root morphology and architecture of *T. chinensis* are highly plastic with respect to changes in the soil water and salt content caused by groundwater salinity (Peng et al., 2022). The root systems of *T. chinensis* with different densities were mainly distributed near the ground surface and expanded outwards onto the beach of the YRD. Low-density *T. chinensis* expands its root growth space mainly by increasing the number of branches, while medium- and high-density *T. chinensis* have fewer branches and strengthen the use of internal resources to reduce competition with neighboring plants (Sun et al., 2021). Song et al. (2017) found that, under different salinity levels, the root–shoot ratio of *T. chinensis* was significantly different, the rooting depth differed, and the root system biomass decreased in a negative logarithmic relationship with increasing soil depth. In conclusion, research on the response of the root system of *T. chinensis* to environmental heterogeneity has mainly focused on root growth and architecture, and there have been few studies of the morphology and function of different orders of fine roots. It is unclear how *T. chinensis* fine roots adapt to heterogeneous groundwater–soil water and salt habitats, which affect the tending and management of low-efficiency forests and the planting and water–salt management of *T. chinensis* seedlings.

We hypothesized that the groundwater level affects the morphological and chemical traits of the fine roots of *T. chinensis* by affecting the content of soil water and salt. Given this background, 7a *T. chinensis* was the subject of research at different groundwater levels formed by microtopographic changes and tidal action in the YRD coastal wetland. To investigate the response and adaptation strategy of the different orders of fine root traits of *T. chinensis* and the trade-off relationship of fine root functional traits under different groundwater levels, this study was expected to provide a theoretical basis and technological support for water–salt management of *T. chinensis* seedlings and improvements in forest stand quality in the YRD.

## Materials and methods

### Study site

The study site is located in the *T. chinensis* Shrub Farm (118°2′–118°4′ E, 38°9′–38°10′ N) in Binzhou Port, Binzhou Beihai New Area, Shandong Province. This area has a continental climate in the temperate East Asian monsoon region. The average temperature is 12.6°C, the average maximum temperature is 31.4°C (July), and the monthly average minimum temperature is −7.99°C (January). The average annual precipitation is 543.2 mm, and it mainly occurs from June to September, accounting for approximately 75% of the total annual precipitation, with less precipitation from November to March. The test area has a shallow groundwater level of 0.5–2.5 m, high salinity, and a flat terrain. The tidal flat soil is alluvial loess parent material. The mechanical composition is mainly silt. The sand is interlaced and easy to compact and has poor air permeability. The vegetation types primarily include shrubs and grasses, among which the shrubs are mainly *T. chinensis*, and the herb distribution includes *Suaeda glauca*, *Suaeda salsa*, *Phragmites australis*, *Imperata cylindrica*, *Setaria viridis*, *Cynanchum chinense*, and *Echinochloa crus-galli* (L.) Beauv.

### Experimental design

In July 2020, a field survey of a *T. chinensis* forest in Binzhou Port, Beihai New Area, Binzhou City, Shandong Province, was conducted, and groundwater level observation tubes were buried. Three PVC pipes with a diameter of 5 cm and a length of 1–3 m were buried in each plot to observe the groundwater level. One end of each PVC pipe was drilled and wrapped with gauze mesh with a diameter of 0.5 mm. The pipes were buried underground, and the groundwater level was measured 24 h later. The groundwater level was measured by a physical water level gauge; the specific groundwater level conditions of each plot are shown in Supplementary Table 1. The groundwater level was divided into five groundwater level conditions: GW1, GW2, GW3, GW4, and GW5. For each groundwater level condition, three quadrats of 10 m × 10 m were randomly set. The mean groundwater levels of GW1, GW2, GW3, GW4, and GW5 were 0.54 ± 0.07, 0.83 ± 0.06, 1.18 ± 0.10, 1.62 ± 0.07, and 2.04 ± 0.05 m, respectively. 7a *T. chinensis* was the subject of this research. The base diameter, plant height, and crown width of each *T. chinensis* specimen were measured in each plot, and one standard tree was selected; i.e., three standard trees were selected at each groundwater level.

### Fine root sample collection and processing

The whole root system containing more than 3 branch grades closest to the standard tree taproot was taken and placed in a liquid nitrogen tank to maintain its activity. The impurities and soil on the surface were removed with low-temperature deionized water before root scanning. According to the Pregitzer root order grading method, the first root of the outer layer is the first level, two first-level roots meet at the second level, two second-level roots meet at the third level, and so on. If different root levels meet, then the higher level is taken as the root level. After the roots were graded, they were placed in labeled containers and stored in a refrigerator for root scanning.

According to the fine root morphology indicator measured by analysis software (Win-RhIZO 2008a) and the measured fine root biomass, the average diameter, SRL, specific surface area (SSA), and RTD of the fine roots were calculated.


$$\mathrm{SRL}\;\left(m/g\right)=\mathrm{Root length}\;\left(m\right)/\mathrm{Root biomass}\;\left(g\right).$$

$$\mathrm{SSA}\;\left({\mathrm{cm}}^{2}/g\right)=\mathrm{Surface area}\;\left({\mathrm{cm}}^{2}\right)/\mathrm{Root biomass}\;\left(g\right).$$

$$\mathrm{RTD}\;\left(g/{\mathrm{cm}}^{3}\right)=\mathrm{Root biomass}\;\left(g\right)/\mathrm{Root volume}\;\left({\mathrm{cm}}^{3}\right).$$

### Fine root nutrients and nonstructural carbohydrate determination

After drying and pulverizing samples of different order-level fine roots, the total C and total N contents were determined by an elemental analyzer (Elementar, vario EL III). The content of soluble sugar and starch was determined by the anthrone colorimetric method, and the nonstructural carbohydrate (NSC) content was the sum of the content of soluble sugar and starch.

### Soil sample collection and determination

Soil samples were collected near the standard trees, and three soil samples were collected from the 0–60 cm soil layer of the concentrated root distribution layer in each plot for the determination of soil water and salt parameters. The soil water content was determined by the oven drying method, the soil salt content was measured by the residue drying method, and the soil to water ratio was 5:1. The absolute concentration of the soil solution (%) = soil salt content (proportion to dry soil mass)/soil water content (proportion to dry soil mass) × 100%.

### Data processing and analysis

Microsoft Office Excel 2010, Origin 2019, and RStudio software applications were used for data processing and mapping. Two-way ANOVA was implemented to detect the effects of GW levels and various fine root orders on fine root traits. When interactions were not significant, independent effects were then considered. When the effects were significant, the LSD test was used to compare the mean values. Data were analyzed with SPSS software, version 26.0. Principal component analysis (PCA) and drawing with Origin 2021. We used the generalized least squares estimation method to fit the measured data to the model. Adequate model goodness of fit was tested by the root-mean-square error of approximation (RMSEA). The statistical results of indirect, direct, and total effects were selected and output to assess groundwater level effects on fine roots using AMOS software (AMOS 23.0 student version). The data in the chart are the mean ± standard deviation.

## Results

### Soil water and salt characteristics

**Figure 1:**
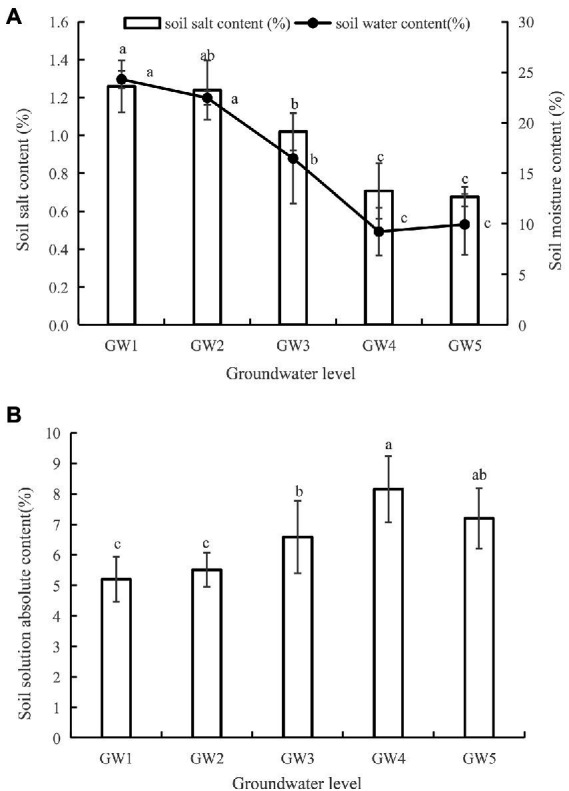
Soil water, salinity **(A)** and soil solution absolute content **(B)** of *Tamarix chinensis* shrubland for different groundwater levels. The lower case letters indicate significant and non-significant differences at *p* < 0.05. The same as below.

With decreasing GW levels, soil salt content generally decreased (Figure 1A). Soil salt contents for GW3, GW4, and GW5 were significantly lower than that for GW1 (1.26%; *p* < 0.05). With decreasing GW levels, the soil water content showed a decreasing trend. There was no statistically significant difference in the soil water content between GW1 and GW2 (*P* > 0.05). The soil water content for GW3, GW4, and GW5 was significantly lower than that for GW1 (*p* < 0.05) by 32.25%, 61.21%, and 59.10%, respectively. With decreasing GW levels, the absolute concentration of the soil solution first increased and then decreased (Figure 1B).

### *Tamarix chinensis* fine root morphology characteristics

#### The effects of groundwater level on root morphological traits

**Figure 2:**
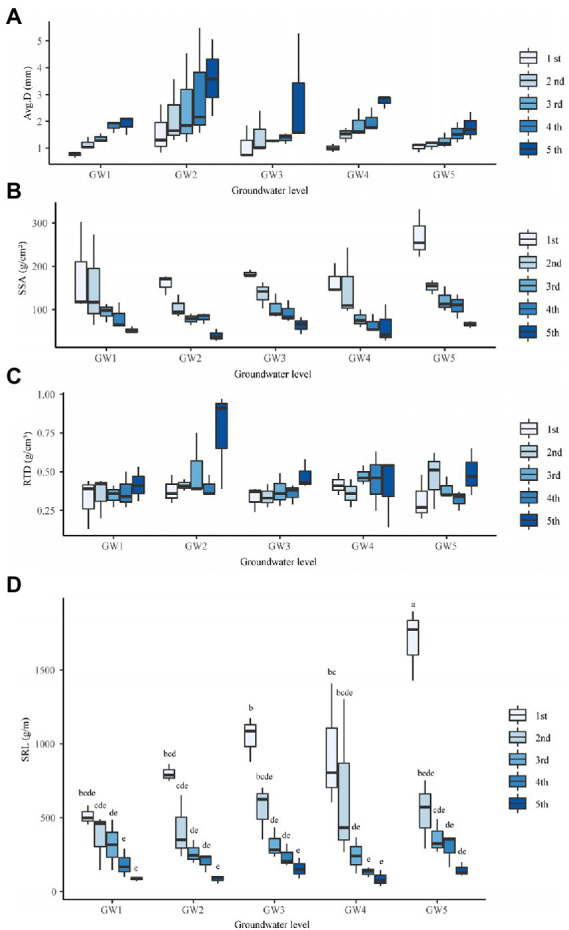
The Avg.D **(A)**, SSA **(B)**, RTD **(C)**, and SRL **(D)** of different orders of fine roots of *Tamarix chinensis* at different groundwater levels. SSA, Specific surface area; RTD, Root Tissue Density; SRL, Specific Root Length.

The GW level had a significant effect on the average diameter of the *T. chinensis* fine roots (*p* < 0.05; Supplementary Table 2). There were no significant differences in the average diameter of the fine roots at GW1, GW3, GW4, and GW5 (*P* > 0.05; Figure 2A). With decreasing GW levels, the average diameters of the first-, second-, third-, fourth-, and fifth-order fine roots all showed increasing, decreasing, increasing, and decreasing trends and reached their maximum for GW2 (Figure 2A).

The GW level had a significant effect on the SSAs of the fine roots of *T. chinensis* (*p* < 0.05; Supplementary Table 2). The SSAs of the fine roots at GW1 and GW2 were significantly lower than those at GW5 (*p* < 0.05) by 35.38% and 27.57%, respectively (Figure 2B). With decreasing GW levels, the SSAs of the third- and fourth-order fine roots showed a decreasing, increasing, and decreasing trend, and the SSAs of the first-, second-, and fifth-order fine roots showed an increasing, decreasing, and increasing trend. They all reached the maximum values for GW5 (Figure 2B).The GW level had no significant effect on the RTDs of *T. chinensis* fine roots (*P* > 0.05; Figure 2C; Supplementary Table 2).

#### Differences in root morphological traits across root orders

Root order had an extremely significant effect on the average diameter, SRL, SSA, and RTD of *T. chinensis* fine roots (*p* < 0.01; Supplementary Table 2). The average diameters of the first-, second-, and third-order roots were significantly lower than those of the fifth-order roots by 57.43%, 42.49%, and 35.49%, respectively (*p* < 0.05; Supplementary Table 2). The SSAs of the second-, third-, fourth-, and fifth-order roots were significantly lower than those of the first-order roots (*p* < 0.05) by 27.30%, 50.16%, 55.14%, and 70.32%, respectively (Supplementary Table 2). The RTDs of the first-, second-, third- and fourth-order roots were significantly lower than those of the fifth-order roots (*p* < 0.05) by 30.60%, 24.15%, 17.64%, and 25.47%, respectively (Supplementary Table 2).

#### The interaction effects of groundwater level and root order on root morphological traits

The interaction of GW level and root order had an extremely significant effect on SRL (*p* < 0.01). At groundwater level GW5, the SRL of first-order roots was significantly higher than that of other groundwater levels and other root orders (*p* < 0.05). At different groundwater levels, with the increase in root order, the specific root length of fine roots showed a downward trend. Under GW5 conditions, the second-, third-, fourth-, and fifth-order fine roots were reduced by 49.06%, 69.95%, 78.99%, and 88.78% compared with the first-order fine roots, respectively (Figure 2D). The interaction effects of GW level and root order on the average diameter, SSA, and RTD were not significant (*p* > 0.05; Supplementary Table 2).

### Nutrient traits of the fine roots of *Tamarix chinensis*

#### Effects of groundwater level on root nutrient traits

**Figure 3:**
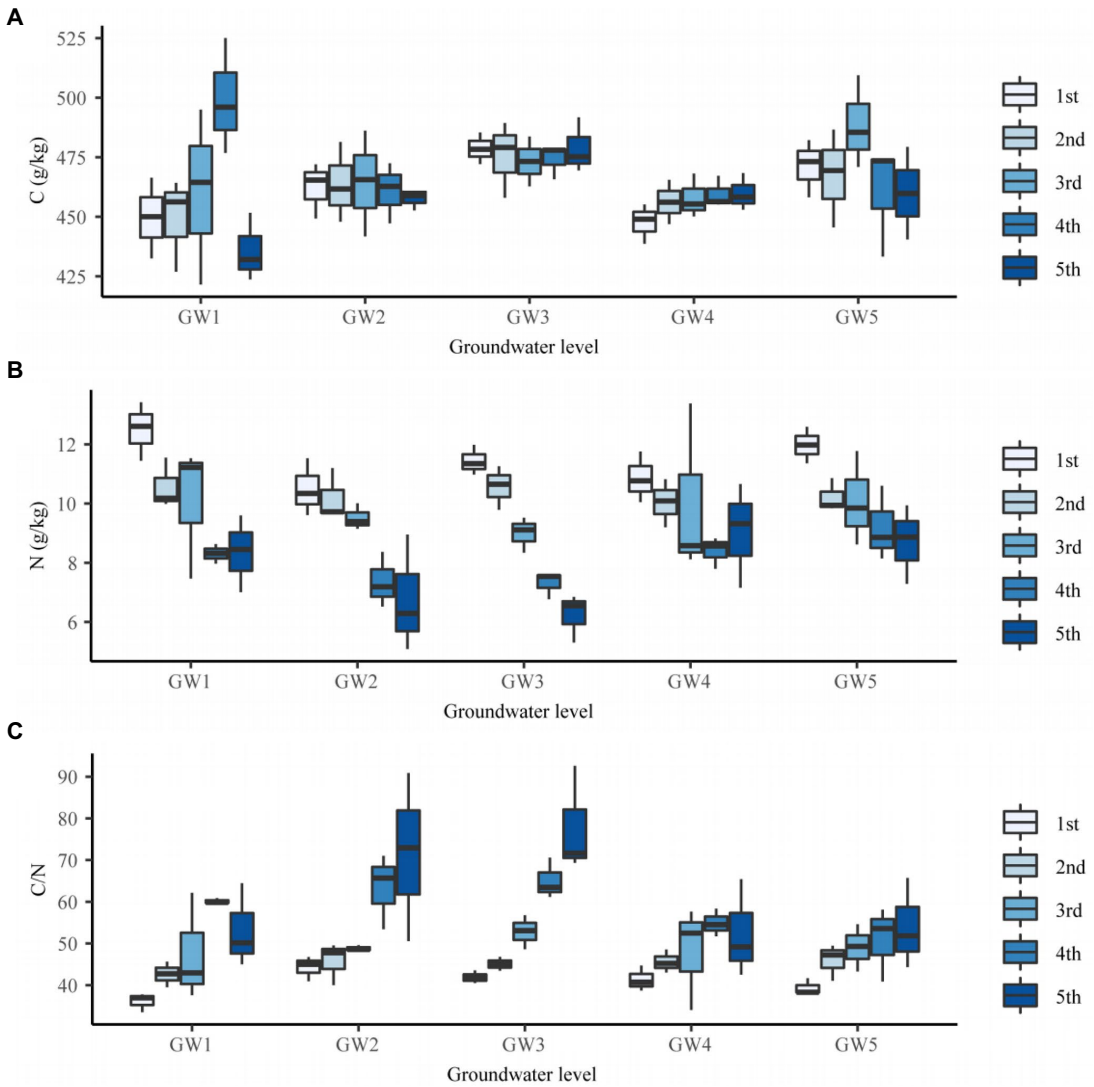
The C content **(A)**, N content **(B)**, and C/N **(C)** of fine roots of different orders of *Tamarix chinensis* at different groundwater levels.

The GW level had a significant effect on the C content of the fine roots of *T. chinensis* (*p* < 0.05; Supplementary Table 3). The C content for GW1, GW2, and GW4 was significantly lower than that for GW3 (*p* < 0.05) by 3.62%, 2.99%, and 4.17%, respectively (Supplementary Table 3). The C content of the fourth-order fine roots reached the maximum value at GW1 (499.31 g/kg; Figure 3A).

The GW level had a significant effect on the N content of *T. chinensis* fine roots (*p* < 0.05; Supplementary Table 3). The N content for GW1 and GW5 was significantly higher than that for GW2 and GW3 (*p* < 0.05), but the difference was not significant with respect to GW4 (*p* > 0.05; Supplementary Table 3). The N content of the first- and second-order fine roots reached the maximum values for GW1, which were 12.50 and 10.57 g/kg, respectively. The N content of the third- and fourth-order fine roots reached maximum values at GW5, which were 10.08 and 9.20 g/kg, respectively. The N content of the fifth-order fine roots reached the maximum value at GW4 (9.04 g/kg; Figure 3B).

The GW level had a significant effect on the C/N ratio of the fine roots of *T. chinensis* (*p* < 0.05; Supplementary Table 3). The C/N values for GW1, GW4, and GW5 were significantly lower than those for GW2 and GW3 (*p* < 0.05; Supplementary Table 3). With decreasing GW levels, the C/N of the first- and fourth-order fine roots reached their maximum values at GW2 (44.23) and GW3 (65.07), respectively (Figure 3C). The C/N ratios of the second-, third-, and fifth-order fine roots reached their maximum values at GW5 (45.92), GW3 (52.82), and GW3 (77.87), respectively (Figure 3C).

#### Differences in root nutrient traits across root orders

Root order had no significant effect on the C content of *T. chinensis* fine roots but had an extremely significant effect on the N content and C/N ratio (*p* < 0.01; Supplementary Table 3). The N content of the second-, third-, fourth- and fifth-order roots was significantly lower than that of the first-order roots (*p* < 0.05) by 9.94%, 14.98%, 29.15%, and 31.70%, respectively (Supplementary Table 3). The C/N values of the second-, third-, fourth- and fifth-order roots were significantly lower than those of the first-order roots (*p* < 0.05; Supplementary Table 3).

The interaction effects of GW level and root order on the C content, N content, and C/N ratio of fine roots were not significant (*p* > 0.05; Supplementary Table 3).

### *Tamarix chinensis* fine root nonstructural carbohydrate traits

#### Effects of groundwater level on root nonstructural carbohydrate traits

**Figure 4:**
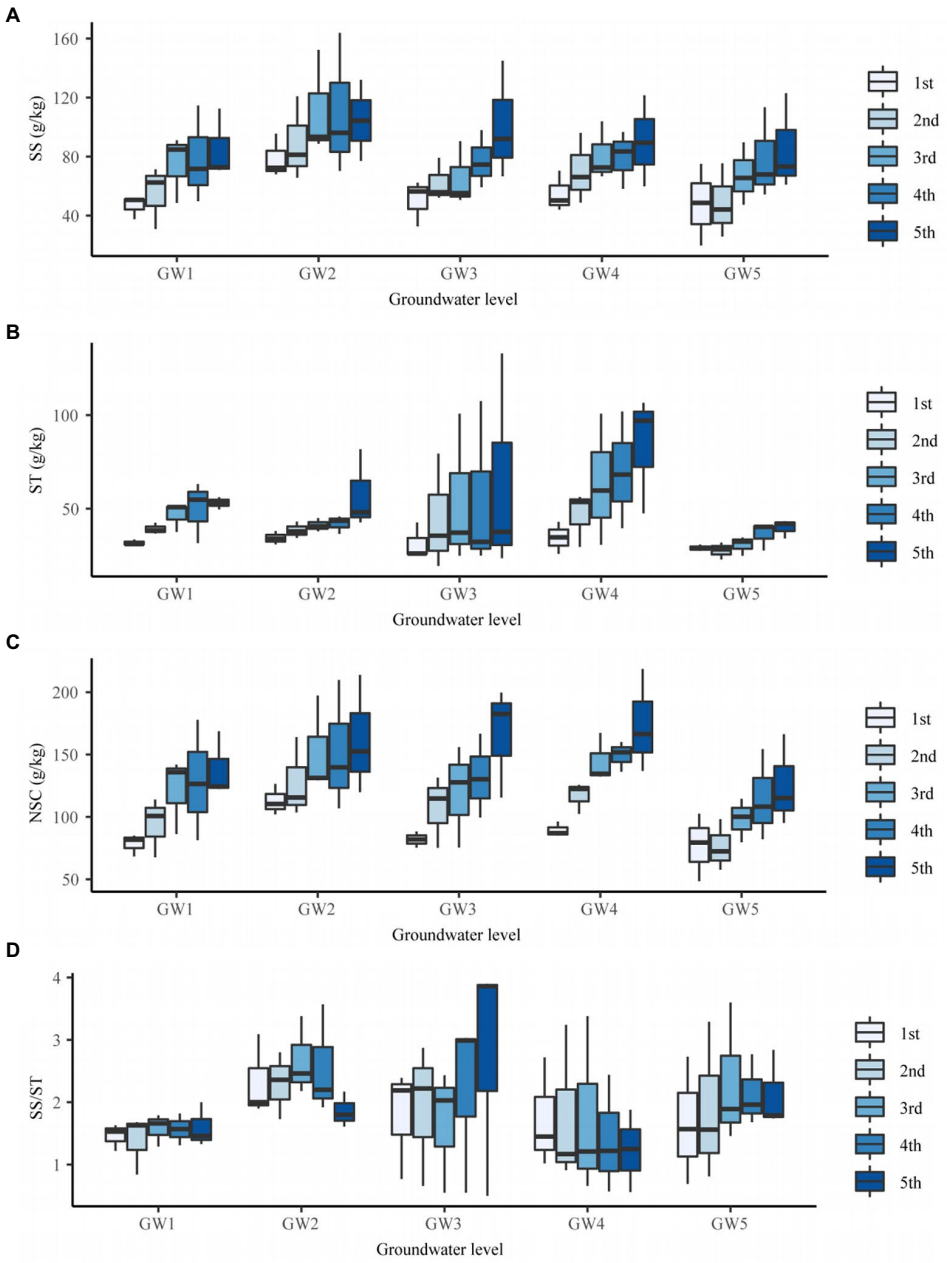
The SS content **(A)**, ST content **(B)**, NSC content **(C)**, and SS/ST **(D)** of fine roots of different order of *Tamarix chinensis* at different groundwater levels. SS, Soluble Sugar; ST, Starch; NSC, nonstructural carbohydrate; SS/ST, Soluble Sugar/Starch.

The GW level had an extremely significant effect on the soluble sugar, starch, and NSC content of *T. chinensis* fine roots (*p* < 0.01) but no significant effect on the soluble sugar/starch content (*p* > 0.05; Supplementary Table 4). The soluble sugar content at GW1, GW3, GW4, and GW5 was significantly lower than that at GW2 (*p* < 0.05) by 31.14%, 27.82%, 23.91%, and 33.62%, respectively (Figure 4A).

At GW1 and GW5, the NSC content of fine roots was significantly lower than that of GW2 (*p* < 0.05) by 20.75% and 30.55%, respectively (Supplementary Table 4). With decreasing GW levels, the NSC content of the first-, second-, third-, and fourth-order fine roots showed an increasing, decreasing, increasing, and decreasing trend, and all reached their maximum values at GW2. The NSC content of the fifth-order fine roots first increased and then decreased and reached its maximum value at GW4 (173.98 g/kg; Figure 4C).

#### Differences in root nonstructural carbohydrate traits across root orders

Root order had a significant effect on the soluble sugar and starch content of *T. chinensis* fine roots (*p* < 0.05) and an extremely significant effect on the NSC content (*p* < 0.01) but no significant effect on the soluble sugar/starch (*p* > 0.05; Supplementary Table 4).

The soluble sugar content of the third-, fourth-, and fifth-order roots was significantly higher than those of the first-order roots (*p* < 0.05; Supplementary Table 4). The soluble sugar content of fine roots at GW2 first increased and then decreased, and that of the third-order roots was the highest (Figure 4A).

The starch content of different order fine roots was between 32.23 and 59.97 g/kg, and the starch content of the third-, fourth-, and fifth-order roots was significantly higher than those of the first-order roots (*p* < 0.05; Supplementary Table 4). With increasing root orders, the soluble sugar content of fine roots at GW1, GW2, GW3, GW4, and GW5 increased, and the soluble sugar content of the fifth-order roots was the highest (Figure 4B).

The NSC content of the third-, fourth-, and fifth-order roots was significantly higher than those of the first-order roots (*p* < 0.05; Supplementary Table 4). With increasing root orders, the NSC content of the fine roots at GW1, GW2, GW3, GW4, and GW5 increased, and the NSC content of fifth-order roots was the highest (Figure 4D).

The interaction effects of groundwater level and root order on the soluble sugar content, starch content, NSC content, and soluble sugar/starch of fine roots were not significant (*p* > 0.05; Supplementary Table 4).

#### Correlation analysis of *Tamarix chinensis* fine root morphology and chemical characteristics and environmental factors

**Figure 5:**
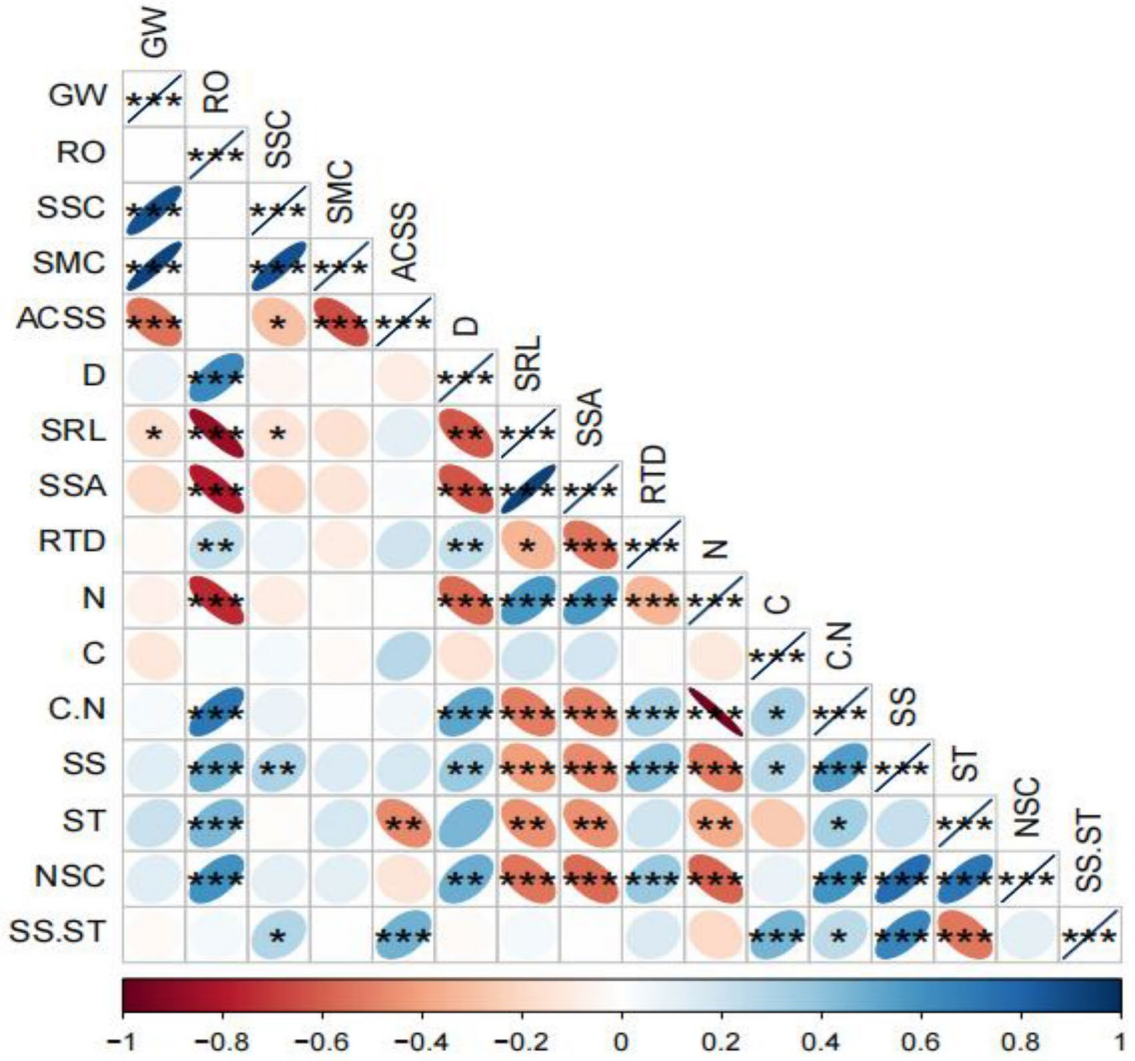
Correlation analysis between environmental factors and the morphology and chemical indices of the fine roots of *Tamarix chinensis.* ^*^p < 0.05; ^**^p < 0.01; ^***^p < 0.001. WG, groundwater level; RO, root order; SSC, soil salinity content; SMC, soil moisture content; ACSS, soil solution absolute content; D, fine root average diameter; SRL, specific root length; SSA, specific surface area; RTD, root tissue density; N, nitrogen; C, carbon; C/N, nitrogen/carbon; SS, soluble sugar; ST, starch; NSC, nonstructural carbohydrate; SS/ST, soluble sugar/starch. The same applies below.

The correlation analysis showed that the groundwater level was significantly negatively correlated with the SRL of *T. chinensis* fine roots (*p* < 0.05). The root order was extremely significantly positively correlated with the average diameter, C/N, and soluble sugar, starch, and NSC content of the fine roots (*p* < 0.01) and was significantly negatively correlated with the SRL, SSA, and N content of the fine roots (*p* < 0.05; Figure 5).

The average diameter was extremely significantly positively correlated with the C/N ratio of the fine roots (*p* < 0.01), significantly positively correlated with the RTD, soluble sugar, and NSC content of the fine roots (*p* < 0.05), extremely significantly negatively correlated with the SSA and N content of the fine roots (*p* < 0.01) and significantly negatively correlated with the SRL of the fine roots (*p* < 0.05). The SRL was extremely significantly positively correlated with the SSA and N content of the fine roots (*p* < 0.01), extremely significantly negatively correlated with the C/N ratio, soluble sugar, and NSC content of the fine roots (*p* < 0.01) and significantly negatively correlated with the starch content of the fine roots (*p* < 0.05). The SSA was extremely significantly positively correlated with the N content of the fine roots (*p* < 0.01), extremely significantly negatively correlated with the RTD, C/N ratio, soluble sugar, and NSC content of the fine roots (*p* < 0.01) and significantly negatively correlated with the starch content of the fine roots (*p* < 0.05). The RTD was extremely significantly positively correlated with the C/N ratio, soluble sugar, and NSC content of the fine roots (*p* < 0.01) and extremely significantly negatively correlated with the NSC content of the fine roots (*p* < 0.01; Figure 5).

The N content was extremely significantly negatively correlated with the C/N, soluble sugar, and NSC content of the fine roots (*p* < 0.01) and significantly negatively correlated with the starch content of the fine roots (*p* < 0.05). The C content was extremely significantly positively correlated with the soluble sugar/starch content of the fine roots (*p* < 0.01) and significantly positively correlated with the C/N ratio and soluble sugar content of the fine roots (*p* < 0.05). The C/N ratio was extremely significantly positively correlated with the soluble sugar and NSC content of the fine roots (*p* < 0.01) and significantly positively correlated with the soluble sugar/starch of the fine roots (*p* < 0.05). The soluble sugar content was extremely significantly positively correlated with the soluble sugar/starch and NSC of the fine roots (*p* < 0.01; Figure 5).

**Figure 6:**
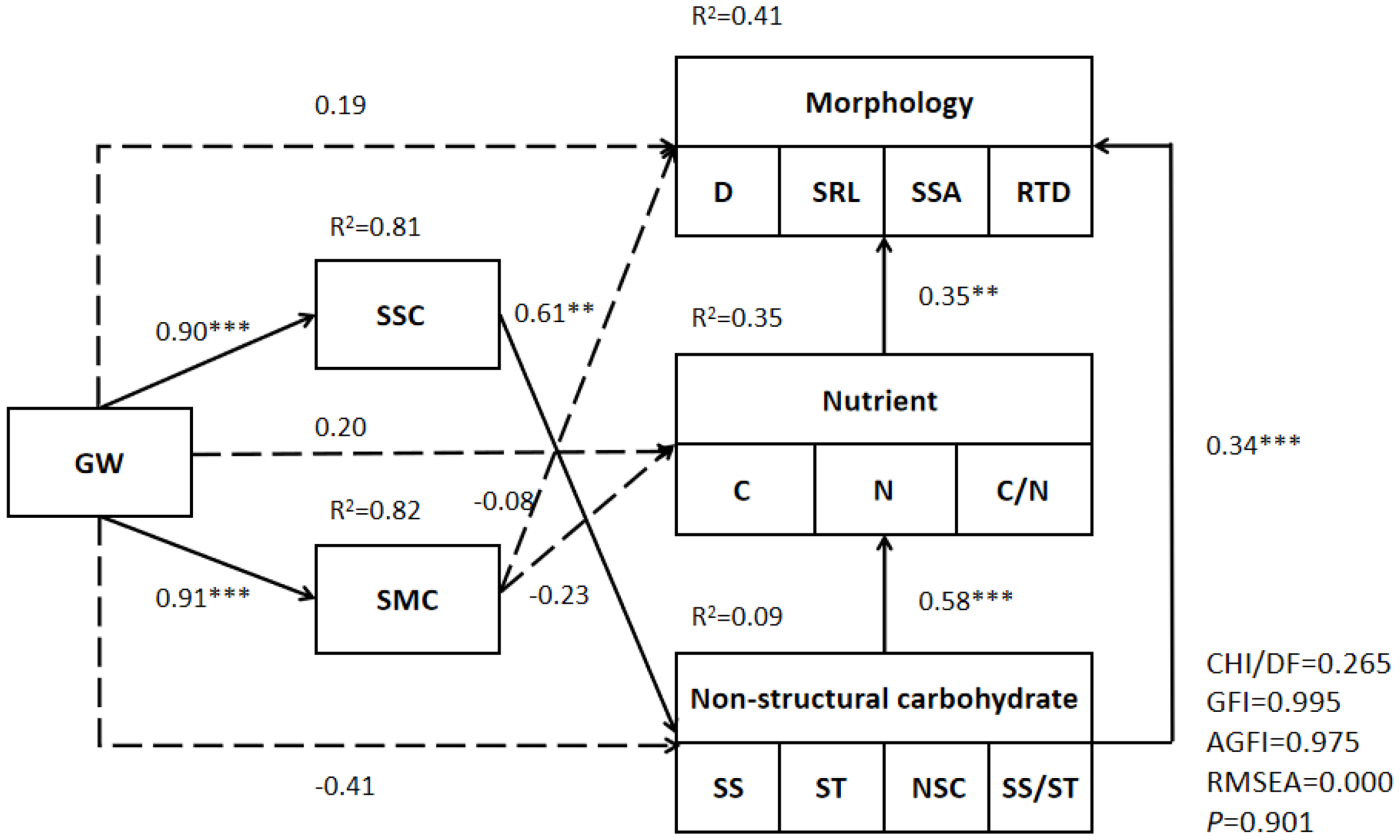
Structural equation model of *Tamarix chinensis* fine root characteristics and environmental factors. **significant at 0.01 level and ***significant at 0.001 level.

A structural equation model was used to adapt and analyze the groundwater level, soil salt content, soil water content, morphology of fine roots, nutrients, and NSC characteristics of *T. chinensis*, and finally, the following structural equation model was established (Figure 6). During modeling, dimension reduction was performed on the morphology (D, SRL, SSA, RTD), nutrients (C, N, C/N), and nonstructural carbohydrates (SS, ST, NSC, SS/ST) of the fine roots. The first-dimension data were extracted, and the interpretation rates were 61.00%, 67.50%, and 53.50%, respectively. The results showed that the structural equation model could explain 41% of the fine root morphology, 35% of the fine root nutrients, and 9% of the fine root nonstructural carbohydrates. Groundwater level had a significantly positive impact on soil salinity and soil water content. Soil salinity had direct and significant positive effects on fine root NSCs, and fine root NSCs had direct and indirect positive effects on morphological characteristics.

## Discussion

### The variations in *Tamarix chinensis* fine root morphology, nutrient, and nonstructural carbohydrate characteristics for different groundwater levels in the coastal wetland

Variation in fine root traits can often be described based on shifts in resource foraging that range from acquisitive (i.e., roots that are inexpensively constructed but that proliferate and acquire resources more rapidly) to conservative (i.e., roots that require greater investment and live longer but acquire resources more slowly). For example, larger diameter roots and higher RTDs are both associated with greater tissue construction costs and longer average fine root life spans, reflecting a more conservative strategy (Caplan et al., 2019). With decreasing groundwater levels, the *T. chinensis* fine root SRL and SSA gradually increased, the RTD gradually decreased, and the average diameter did not undergo an obvious regular change. This outcome indicated that the high groundwater levels of GW1 (0.54 m) and GW2 (0.81 m) were relatively rich in soil salt-based ions compared with the low groundwater levels of GW4 (1.62 m) and GW5 (2.04 m); A relatively high soil salinity generally causes osmotic stress in plants and disrupts their nutrient ion balance, thereby affecting physiological and biochemical processes such as growth, osmotic adjustment synthesis, and lipid metabolism (Zhang et al., 2017), and ultimately limits the growth rate of the fine roots; the SSA and SRL of the fine roots were low; the RTD was high; and the nutrient absorption rate per unit of dry weight of fine roots was low (Xu et al., 2011). At the low groundwater levels of GW4 (1.62 m) and GW5 (2.04 m), *T. chinensis* improved its water use efficiency by increasing the SSA and SRL of the fine roots and accelerated growth turnover by reducing the RTD of the fine roots.

Increasing the SSA and SRL of the fine roots at the low groundwater levels of GW4 (1.62 m) and GW5 (2.04 m) can improve the water utilization efficiency of *T. chinensis* by reducing the RTD of fine roots to accelerate growth turnover, reduce water loss, and improve the absorption of water. However, studies have shown that, for the conditions of high groundwater levels in the sinking zone of Shengjin Lake in the Tongjiang Lake wetland, the SRL and RTD of *Carex thunbergii* fine roots were significantly reduced, while the average diameter and SSA were significantly increased. The difference between this study of *C. thunbergii* and the results for the average diameter of fine roots may occur because wetland plants have more developed ventilation tissues, which can expand the diameter of their air cavities to increase oxygen acquisition under hypoxic stress caused by high groundwater level conditions, significantly affecting the morphological structure characteristics of fine roots (Yuan and Huangpu, 2020). The decrease in groundwater level had no significant effect on the root length or root surface area of *Haloxylon ammodendron* in the arid desert areas, but the SRL decreased significantly (Liu et al., 2022). Short-term water treatment significantly affected the different orders of fine root average diameter, SRL, and RTD of *Machilus pauhoii*, and drought stress significantly increased the diameter of the third-order fine roots and decreased the RTD of the first- and second-order fine roots (Zou et al., 2018). *H. ammodendron*, *M. pauhoii*, and *T. chinensis* are all woody plants. Due to their dissimilar tolerance characteristics, their fine root morphology changes in response to water also showed great differences with the variety of species and habitat conditions.

The content of C and N in fine roots is an important part of nutrient cycling and energy flow in the ecosystem and an index for measuring the cost of fine root biomass construction and maintenance. The C content of fine roots is associated with construction costs (Withington et al., 2006; Terzaghi et al., 2013), whereas the N content is associated with metabolic activity, respiration, and root longevity (Withington et al., 2006; McCormack et al., 2012). As a consequence, the C/N ratio can indicate the life span of fine roots (Withington et al., 2006), i.e., the higher the fine root C/N ratio, the longer the life span, and the lower the fine root turnover rate (Pregitzer et al., 2002; McCormack et al., 2012). With the decrease in GW levels, the C content of fine roots of *T. chinensis* in coastal beaches did not show an obvious change trend, while the N content first decreased and then increased, and the C/N ratio first increased and then decreased. At the groundwater level of GW3 (1.18 m), the soil water and salt conditions of the *T. chinensis* forest were relatively suitable, the respiration of the fine roots of *T. chinensis* decreased, and the fine root life increased. Under relatively high and low groundwater levels, the fine roots had higher N content and lower C/N ratios, it could improve its adaptability to high salinity and low water content soil environments by increasing the turnover rate of fine roots and enhancing their metabolism (Di et al., 2018).

Nonstructural carbohydrates are composed of starch and soluble sugar, which are synthesized by plant leaves through photosynthesis and transported to the root system. Nonstructural carbohydrates not only can provide raw materials for building root structures but also are important substances that enable a root system to resist stress related to adversity (Zhang et al., 2020). With decreasing groundwater levels, the soluble sugar, starch, and NSC content and the soluble sugar/starch of *T. chinensis* fine roots first increased and then decreased. The soil salt content was higher at the high groundwater level, but the NSC characteristics of fine roots did not respond in a sensitive manner to it. It was previously assumed that a high groundwater level, GW1 (0.54 m), increased the soluble sugar content and soluble sugar/starch ratio of the fine roots of *T. chinensis* and increased the osmotic potential of the cell solution to resist physiological stress due to drought, which was caused by high concentrations of salt ions. However, the study found that the soluble sugar content and soluble sugar/starch ratio of GW1 (0.54 m) were low under high groundwater level conditions, which may have been due to the dilution of soil salts by high soil water content for high groundwater level conditions, resulting in the low absolute concentrations of soil solution or the limited adaptability of *T. chinensis* fine roots to salt stress. Further experimental studies are needed to investigate the specific reasons. For the GW5 (2.04 m) low groundwater level, the starch and NSC content of the fine roots of *T. chinensis* was the lowest, but the soluble sugar/starch ratio was higher. This result could have been due to the stronger growth and photosynthesis ability of *T. chinensis* at low groundwater levels and the increased amount of photosynthetic products (Xia et al., 2018). However, this result may have been due to the low soil water content at the GW5 (2.04 m) low groundwater level and the rapid growth of plant roots in a short time to obtain sufficient water. To meet the needs of respiratory metabolism, NSCs are more involved in physiological activities, such as metabolism, in the form of soluble sugar, and the amount of starch converted into soluble sugar is greater than the consumption of soluble sugar (Meghan and Robin, 2020). It may be that *T. chinensis* can meet its water demand by increasing the soluble sugar content in its fine roots and increasing the osmotic potential of cells under the condition of relatively scarce water. Under mild drought stress, the changes in soluble sugar content in the roots of *Caragana microphylla* in Khorchin Sandy Land were similar to the results of this study. With the increase in drought stress, the starch content of *C. microphylla* increased to synthesize defensive substances and resist drought-related stress (Lei et al., 2017). The specific root respiration and NSC content of *Cunninghamia lanceolata* did not change under the condition of isolated precipitation, but the starch content of fine roots with diameters of 1–2 mm increased, and the soluble sugar content and soluble sugar/starch ratio decreased significantly, showing that *C. lanceolata* responded to drought-related stress caused by reduced precipitation by increasing the proportion of NSC storage (Zhong et al., 2016). However, the changes in starch content in the fine roots of *T. chinensis* in coastal wetlands were not consistent with the changes in the above two drought-related stress conditions, which simulated drought-induced stress conditions. In this study, the groundwater level was extremely significantly positively correlated with the soil salt content (*p* < 0.01), so the changes in soil water and salt conditions caused by groundwater level affected the fine root starch content of *T. chinensis*. Additionally, due to the different plant species and degrees of soil moisture reduction, the research results were inconsistent. However, Zheng et al. (2014) also found that the increase in NSCs in plants was not related to resistance to drought-induced stress, but the lack of water in plants affected the structural construction of plant tissues when the starch content increased as the premise because starch is a nonpermeable active substance. Therefore, there should be some differences in the responses of physiological and biochemical processes of plants to water-related stress.

The structural equation model showed that soil salinity had a significant, positive effect on the NSCs in the fine roots of *T. chinensis*. Fine root NSCs had direct significant effects on fine root nutrients and fine root morphology. Fine root nutrients had an indirect effect on morphology, while soil moisture had no significant effect on fine root indicators. It was concluded that the groundwater–soil salt conditions played a major role in the fine roots of *T. chinensis* in the groundwater–soil salt system, and water was a secondary factor. In arid regions, groundwater mainly affects the growth of plant roots through the soil water content, and the water deficit is the limiting factor. The groundwater level in the YRD is high, and water is not the main limiting factor. However, since the rise in groundwater level will aggravate the degree of soil salinization, salinity is the main factor affecting the fine roots of *T. chinensis*. Fine root morphology and function coordinate to adapt to changes in groundwater and soil water and salt. *T. chinensis* is a typical halophyte. Under salt stress, osmotic adjustment substances, such as NSCs, increase; fine root nutrient content increases; and stress resistance is enhanced. Fine root morphology is adjusted accordingly to improve root construction costs. With the increase in stress, *T. chinensis* fine roots changed from an acquisition strategy to a conservative strategy. In summary, there was not a single response of the fine root morphology and functional indicators of *T. chinensis* to groundwater levels, but the fine root morphology, chemical characteristics, and other aspects of water and salt environment heterogeneity showed a synergistic response, and there were trade-offs and adjustments.

### The variation in *Tamarix chinensis* fine root morphology, nutrient, and nonstructural carbohydrate characteristics of different orders in the coastal wetland

Morphological plasticity is a manifestation of plant survival strategies in heterogeneous habitats. Fine roots have high morphological and functional heterogeneity at the root-order level (Kong et al., 2019). Affected by the order of growth and development, the root traits related to plant function change strongly and nonlinearly with increasing root orders, reflecting the functional transformation from resource absorption to transportation (Pregitzer et al., 2002; McCormack et al., 2015). In this study, with increasing fine root orders, the average diameter and RTD of fine roots increased, the SRL and SSA decreased, the C/N ratio gradually increased, the N content gradually decreased, and the soluble sugar, starch, and NSC content gradually increased. For example, Yu et al. (2021) studied the morphological characteristics of the first five fine roots of *Pinus koraiensis* at different latitudes in China, Pregitzer et al. (2002) studied the first three fine roots of nine coniferous and broad-leaved species in North America, and Shi (2008) studied the first five fine roots of 20 broad-leaved species in the natural secondary forest of Maoer Mountain in Northeast China, all of which revealed similar changes to this study, further confirming that there were certain functional differences between fine roots of different orders. The morphological structural heterogeneity of fine roots follows a general rule in plants. The root order method can distinguish the heterogeneity of fine roots (Pregitzer et al., 2002). First- and second-order fine roots mainly provide nutrient acquisition in the root system, without secondary growth processes, short life cycles, strong metabolic activity, and fast respiration rates, and they consume more NSCs. Therefore, they have higher SRLs, SSAs, and N content, increase land use area with less carbon input, and meet the needs of plants with high metabolic rates for nutrient resources (Chen et al., 2017). Third-, fourth-, and fifth-order fine roots have secondary growth processes, large diameters, and high RTDs, and they can better undertake nutrient transport and storage functions (Guo et al., 2008; McCormack et al., 2015); therefore, they also have a higher NSC content.

## Conclusion

With decreasing groundwater levels, the soil salinity and water content gradually decreased in the coastal wetland of the YRD. Changes in soil salinity caused by groundwater levels in this region are the main factors affecting fine roots. The fine roots of *T. chinensis* in the coastal wetland of the Yellow River Delta responded to the heterogeneity of different groundwater–soil water–salt systems through trade-offs and changes in morphological, nutrient, and chemical characteristics. With decreasing groundwater levels, the specific root length, specific surface area, and root tissue density of fine roots of *T. chinensis* increased gradually, indicating that the growth rate of fine roots was slower at a high groundwater level than at a low groundwater level. *T. chinensis* increased the opportunity for root-specific surface area to improve water absorption at a low groundwater level. Under a medium groundwater level, the content of N and C/N decreased, and the content of soluble sugar, starch, and nonstructural carbohydrates increased to inhibit the respiration of fine roots, and the utilization of soluble sugar and starch was lower. At high groundwater and low groundwater levels, the fine roots of *T. chinensis* must enhance their metabolism and improve their adaptability to environments with high salt and low groundwater content. The fine roots of *T. chinensis* at different orders were significantly different in morphology, nutrients, and chemical characteristics, and the morphological changes of fine roots were compatible with their functions.

## Data availability statement

The raw data supporting the conclusions of this article will be made available by the authors, without undue reservation.

## Author contributions

JS contributed to writing—original draft, writing—review and editing, investigation, and data curation. JX contributed to supervision and funding acquisition. PS contributed to methodology and investigation. JM, FG, and YL contributed to investigation and data curation. XX contributed to investigation and validation. MD contributed to investigation and analysis. CL contributed to writing—review and editing and methodology. All authors contributed to the article and approved the submitted version.

## Funding

This research was financially supported by the Joint Funds of the National Natural Science Foundation of China (no. U2006215), the Forestry Science and Technology Innovation Project of Shandong Province (no. 2019LY006), the Basic Investigation Project for the Development Quality of Wetland Park in Shandong Province (no. SDGP202202001808A_001), and the Taishan Scholars Program of Shandong Province, China (no. TSQN201909152).

## Conflict of interest

The authors declare that the research was conducted in the absence of any commercial or financial relationships that could be construed as a potential conflict of interest.

## Publisher’s note

All claims expressed in this article are solely those of the authors and do not necessarily represent those of their affiliated organizations, or those of the publisher, the editors and the reviewers. Any product that may be evaluated in this article, or claim that may be made by its manufacturer, is not guaranteed or endorsed by the publisher.
