# Supplementary material for: Response of the fine root morphological and chemical traits of Tamarix chinensis to water and salt changes in coastal wetlands of the Yellow River Delta
Source: Front Plant Sci. 2022 Oct 11;13:952830. doi: 10.3389/fpls.2022.952830 (PMC9592973; doi:10.3389/fpls.2022.952830)
Supplement: Supplementary file 1 [file Data_Sheet_1.docx]

**Table 1. Overview of sample sites in the coastal wetland**

| Sample plots | Latitude (E) | Longitude (N) | Groundwater level | Dominant species |
| --- | --- | --- | --- | --- |
| GW1-1 | 118°3′42.00″ | 38°10′11.00″ | 0.47 m | *S. Salsa*, *T. chinensis* |
| GW1-2 | 118°3′30.00″ | 38°10′5.00″ | 0.55 m | *S. Salsa*, *T. chinensis* |
| GW1-3 | 118°3′52.00″ | 38°10′1.50″ | 0.60 m | *S. Salsa*, *T. chinensis* |
| GW2-1 | 118°3′30.00″ | 38°10′11.92″ | 0.78 m | *S. Salsa*, *T. chinensis* |
| GW2-2 | 118°3′38.54″ | 38°9′54.70″ | 0.82 m | *S. Salsa*, *T. chinensis* |
| GW2-3 | 118°3′50.00″ | 38°9′52.70″ | 0.90 m | *C. Chinense*, *T. chinensis* |
| GW3-1 | 118°3′29.00″ | 38°10′21.00″ | 1.10 m | *S. Salsa*, *T. chinensis* |
| GW3-2 | 118°3′57.00″ | 38°10′21.00″ | 1.30 m | *S. Salsa*, *T. chinensis* |
| GW3-3 | 118°3′29.00″ | 38°9′57.00″ | 1.15 m | *P. australis*, *T. chinensis* |
| GW4-1 | 118°3′17.00″ | 38°10′22.94″ | 1.58 m | *S. Salsa*, *T. chinensis* |
| GW4-2 | 118°3′20.00″ | 38°10′13.00″ | 1.70 m | *S. Salsa*, *T. chinensis* |
| GW4-3 | 118°3′21.00″ | 38°9′58.00″ | 1.70 m | *C. Chinense*, *T. chinensis* |
| GW5-1 | 118°3′59.00″ | 38°10′18.00″ | 2.03 m | *P. australis*, *T. chinensis* |
| GW5-2 | 118°3′20.06″ | 38°10′6.02″ | 2.10 m | *P. australis*, *T. chinensis* |
| GW5-3 | 118°3′40.00″ | 38°10′3.00″ | 2.00 m | *S. Salsa*, *T. chinensis* |

**Table 2. The influence of groundwater level, root order and their interaction on the morphological characteristics in fine roots of *T. chinensis***

| Treatment | | Average diameter（mm） | Specific root length（m/g） | Specific surface area（cm^2^/g） | Tissue density  （g/cm^3^） |
| --- | --- | --- | --- | --- | --- |
| Groundwater level | GW1 | 1.39±0.48b | 2.93±1.64c | 92.89±44.21b | 0.49±0.16a |
|  | GW2 | 2.59±0.79a | 3.52±2.77bc | 104.10±49.73b | 0.42±0.04ab |
|  | GW3 | 1.60±0.68b | 4.62±3.59b | 116.07±45.40ab | 0.40±0.08ab |
|  | GW4 | 1.83±0.64b | 4.14±3.73bc | 111.45±51.91ab | 0.36±0.03b |
|  | GW5 | 1.35±0.32b | 6.08±4.26a | 143.73±76.92a | 0.38±0.06b |
| Root order | 1 | 1.09±0.30c | 9.98±4.39a | 191.27±44.76a | 0.35±0.04b |
|  | 2 | 1.48±0.42bc | 5.09±1.20b | 139.05±20.25b | 0.39±0.05b |
|  | 3 | 1.66±0.55bc | 3.00±0.47c | 95.34±18.49c | 0.42±0.07ab |
|  | 4 | 1.96±0.66ab | 2.10±0.60cd | 85.80±15.98cd | 0.38±0.05b |
|  | 5 | 2.57±0.74a | 1.12±0.35d | 56.78±10.84d | 0.51±0.14a |
| F value | Groundwater level | 5.04^*^ | 6.27^**^ | 2.97^*^ | 2.18^ns^ |
|  | Root order | 6.03^**^ | 53.89^**^ | 22.78^**^ | 2.94^**^ |
|  | Groundwater level×Root order | 0.28^ns^ | 3.10^**^ | 0.55^ns^ | 0.78^ns^ |

Note: Different lowercase letters in the same column indicate significant differences between treatments. The same below

**Table 3. The influence of groundwater level, root order and their interaction on the C and N content**

**in fine roots of *T. chinensis***

| Treatment | | C | N | C/N |
| --- | --- | --- | --- | --- |
| Groundwater level | GW1 | 458.85±24.23bc | 9.96±1.74a | 47.92±9.28b |
|  | GW2 | 461.83±2.59bc | 8.87±1.69b | 54.73±12.04a |
|  | GW3 | 476.07±2.55a | 8.91±2.17b | 56.55±14.90a |
|  | GW4 | 456.23±5.04c | 9.67±0.96ab | 48.45±5.36b |
|  | GW5 | 469.45±11.70ab | 10.03±1.25a | 47.84±5.54b |
| Root order | 1 | 461.85±13.42 | 11.45±0.81a | 40.60±3.05c |
|  | 2 | 462.34±10.13 | 10.31±0.24b | 45.00±1.36c |
|  | 3 | 468.92±12.45 | 9.74±0.48b | 49.26±2.08b |
|  | 4 | 470.86±17.01 | 8.11±0.79c | 58.86±5.93a |
|  | 5 | 458.46±15.26 | 7.82±1.24c | 61.77±12.00a |
| F value | Groundwater level | 3.855^*^ | 2.885^*^ | 4.604^*^ |
|  | Root order | 5.026^ns^ | 3.262^**^ | 10.140^**^ |
|  | Groundwater level×Root order | 0.174^ns^ | 0.225^ns^ | 0.185^ns^ |

**Table 4. The influence of groundwater level, root order and their interactions on non-structural carbohydrates in fine roots of *T. chinensis***

| Treatment | | [Soluble](javascript:;) [sugar](javascript:;)  （g/kg） | Starch  （g/kg） | NSC  （g/kg） | [Soluble](javascript:;) [sugar](javascript:;)/  Starch |
| --- | --- | --- | --- | --- | --- |
| Groundwater  level | GW1 | 68.03±16.55b | 44.10±8.59ab | 112.12±25.08bc | 1.52±0.09a |
|  | GW2 | 98.78±14.31a | 42.70±8.83ab | 141.49±20.41a | 2.35±0.31a |
|  | GW3 | 71.30±19.23b | 50.04±12.54ab | 121.34±31.07abc | 2.06±0.43a |
|  | GW4 | 75.17±13.37b | 59.69±19.41a | 134.86±32.52ab | 1.58±0.25a |
|  | GW5 | 65.58±17.24b | 35.69±5.16b | 98.27±22.20c | 2.03±0.25a |
| Root order | 1 | 55.63±13.24c | 32.23±2.27c | 87.86±14.83d | 1.79±0.32a |
|  | 2 | 65.04±15.82bc | 39.33±7.39bc | 104.37±20.09cd | 1.85±0.33a |
|  | 3 | 80.02±18.63ab | 47.27±12.60abc | 127.29±21.76bc | 2.00±0.48a |
|  | 4 | 84.77±14.18ab | 50.60±12.94ab | 135.38±15.44ab | 1.97±0.48a |
|  | 5 | 93.40±8.91a | 59.79±16.18a | 153.18±20.34a | 1.91±0.57a |
| F value | Groundwater level | 3.855^*^ | 2.885^*^ | 4.604^*^ | 2.107^ns^ |
|  | Root order | 5.026^*^ | 3.262^*^ | 10.140^**^ | 0.125^ns^ |
|  | Groundwater level×Root order | 0.174^ns^ | 0.225^ns^ | 0.185^ns^ | 0.328^ns^ |
